# Three year results of Blessed: Expanded access for DeltaRex-G for an intermediate size population with advanced pancreatic cancer and sarcoma (NCT04091295) and individual patient use of DeltaRex-G for solid malignancies (IND# 19130)

**DOI:** 10.3389/fmmed.2022.1092286

**Published:** 2022-12-16

**Authors:** Sant P. Chawla, Steven Wong, Doris Quon, Ania Moradkhani, Victoria S. Chua, Don A. Brigham, Rebecca A Reed, William Swaney, Frederick L. Hall, Erlinda M. Gordon

**Affiliations:** ^1^ Cancer Center of Southern California/Sarcoma Oncology Center, Santa Monica, CA, United States; ^2^ Aveni Foundation, Santa Monica, CA, United States; ^3^ Expression Therapeutics, Tucker, GA, United States

**Keywords:** DeltaRex-G, cancer gene therapy, CCNG1, cyclin G1 inhibitor, pancreatic cancer, sarcoma, breast cancer, basal cell carcinoma

## Abstract

**Background:** Innovative treatments are urgently needed for metastatic cancer. DeltaRex-G, a tumor-targeted retrovector encoding a dominant-negative/cytocidal cyclin G1 (CCNG1 gene) inhibitor construct—has been tested in over 280 cancer patients worldwide in phase 1, phase 2 studies and compassionate use studies, demonstrating long term (>10 years) survivorship in patients with advanced cancers, including pancreatic cancer, osteosarcoma, malignant peripheral nerve sheath tumor, breast cancer, and B-cell lymphoma.

**Patient and Methods:** Endpoints: Survival, response, treatment-related adverse events. Study one is entitled “Blessed: Expanded Access for DeltaRex-G for Advanced Pancreatic Cancer and Sarcoma (NCT04091295)”. Study two is entitled “Individual Patient Use of DeltaRex-G for Solid Malignancies (Investigational New Drug#19130). In both studies, patients will receive DeltaRex-G at 1-3 x 10e11 cfu i.v. over 30–45 min, three x a week until significant disease progression or unacceptable toxicity or death occurs.

**Results:** Seventeen patients were enrolled, nine sarcoma, two pancreatic adenocarcinoma, one non-small cell lung cancer, two breast carcinoma, one prostate cancer, one cholangiocarcinoma and one basal cell carcinoma and actinic keratosis. Three patients were enrolled in Study 1 and 14 patients were enrolled in Study 2. Twelve of 17 enrolled patients were treated with DeltaRex-G monotherapy or in combination with United States Food and Drug Administration-approved cancer therapies. Five patients died before receiving DeltaRex-G. Efficacy Analysis: Of the 12 treated patients, 5 (42%) are alive 15–36 months from DeltaRex-G treatment initiation. Two patients with early-stage HR + HER2+ positive or triple receptor negative invasive breast cancer who received DeltaRex-G as adjuvant/first line therapy are alive in complete remission 23 and 16 months after DeltaRex-G treatment initiation respectively; three patients with metastatic chordoma, chondrosarcoma and advanced basal cell carcinoma are alive 36, 31, and 15 months after DeltaRex-G treatment initiation respectively. Safety Analysis: There were no treatment-related adverse events reported.

**Conclusion:** Taken together, the data suggest that 1) DeltaRex-G may evoke tumor growth stabilization after failing standard chemotherapy, 2) DeltaRex-G may act synergistically with standard chemotherapy/targeted therapies, and 3) Adjuvant/first line therapy with DeltaRex-G for early-stage invasive carcinoma of breast may be authorized by the USFDA when patients refuse to receive toxic chemotherapy.

## 1 Introduction

Innovative therapies that address fundamental drivers of cancer are desperately needed to counteract the invariable fatal outcome of progressive metastatic disease. Defects in executive cell cycle activation and control mechanisms are among the proximal oncogenic drivers, thus the targeting of deregulated cell cycle control elements has emerged as a major regulatory theme and therapeutic strategy (Gordon et al., 2018). Interventional cell cycle inhibitor therapy, exemplified by DeltaRex-G—a tumor-targeted retrovector encoding a cytocidal “dominant-negative” (dnG1) expression construct of the cyclin G1 (CCNG1 oncogene); dnG1 expression blocks cell-activation, transcription control, and survival functions of the cyclin G1/Cdk/myc/Mdm2/P53 axis. Demonstrably capable of inducing apoptosis in proliferative tumor cells and supportive neovasculature in the presence or absence of a functional p53 locus (TS53 tumor suppressor gene), DeltaRex-G exhibits broad spectrum clinical utilty in numerous tumor types. DeltaRex-G has been tested in over 280 cancer patients worldwide in phase 1 and 2 studies, inducing long term (>10-year) survivorship in patients with intractable metastatic cancer including pancreatic cancer, osteosarcoma, soft tissue sarcoma, breast cancer, and B-cell lymphoma (Kim et al., 2017; Al-Shihabi et al., 2018; Gordon et al., 2018; Liu et al., 2021). Hence, further clinical development and expanded access of DeltaRex-G is recommended for cancer patients who have few or no therapeutic options.

### 1.1 DeltaRex-G (former names: Mx-dnG1, dnG1 or Rexin-G)

**FIGURE 1:**
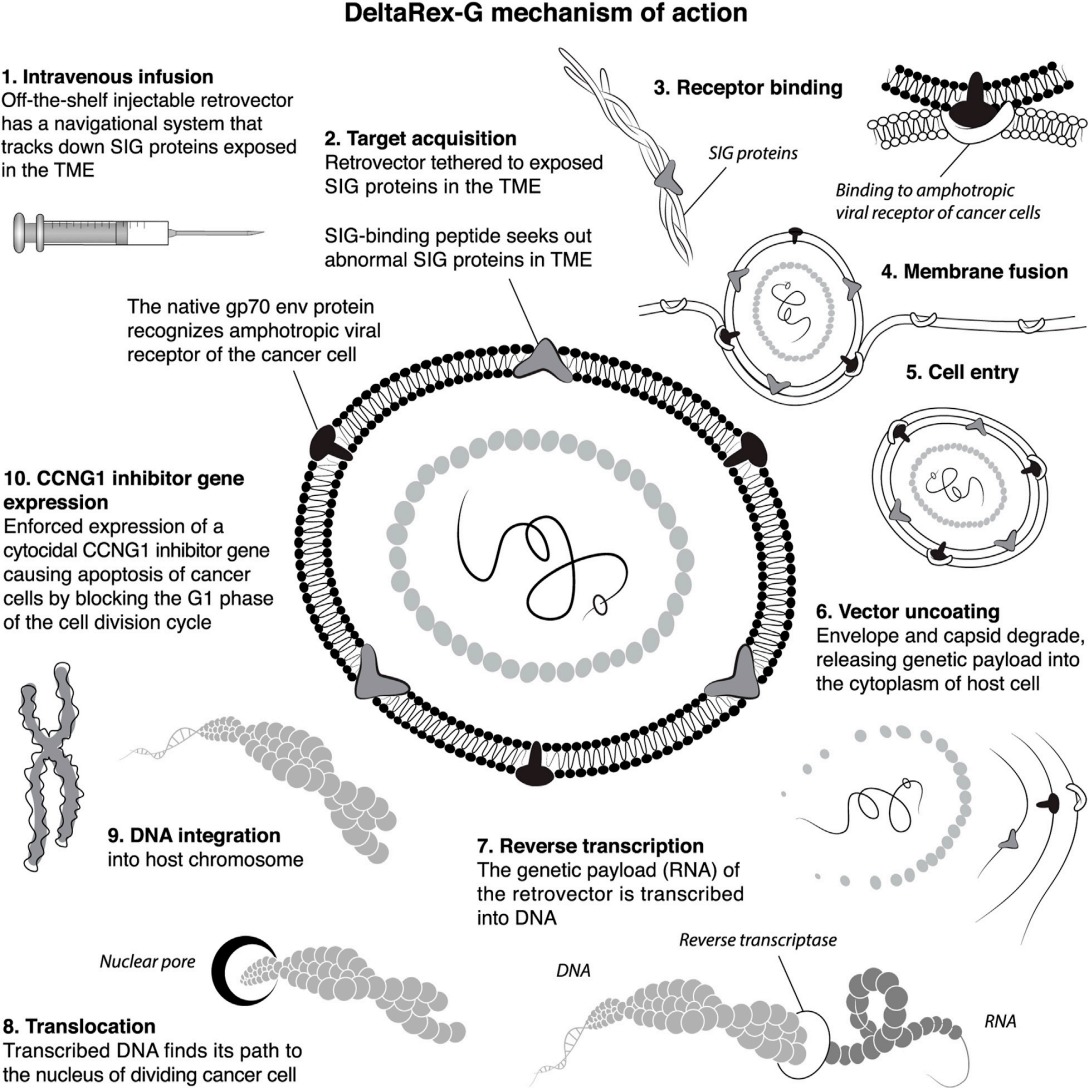
A Diagramatic ten-step illustration of DeltaRex-G tumor targeting mechanisms of action. The DeltaRex-G nanoparticle displays a pan-collagenous SIG-binding peptide derived from vWF coagulation factor on its gp70 envelope protein. When injected i.v., DeltaRex-G seeks out tumors and accumulates in cancerous lesions by binding to abnormal SIG proteins exposed in the TME as a result of tumor invasion processes. This targeted retrovector has the innate property of MLV binding to the natural amphotropic cell receptor, fusing, entering, uncoating and integrating into the chromosomes of actively dividing cells only (i.e., cancer cells), while sparing normal cells. DeltaRex-G bears a cytocidal dominant-negative Cyclin G1 inhibitory construct, which causes cell death through apoptosis. *CCNG1*, cyclin G1; SIG, abnormal signature proteins; vWF, von Willebrand factor; TME, tumor microenvironment (Morse et al., 2021).

DeltaRex-G is a tumor targeted, murine leukemia virus (MLV)-based retrovector 1) displaying a Signature-pan-collagen binding decapeptide on its gp70 envelope protein and 2) encoding a truncated N-terminal deletion mutant construct of the human CCNG1 oncogene under the control of a hybrid LTR/CMV promoter (Figure 1). The DeltaRex-G expression vector also contains the neomycin resistance (neo^r^) gene which is driven by the SV40 early promoter. The DeltaRex-G vector is produced by transient co-transfection of three plasmids in 293T (human kidney 293 cells transformed with SV40 large T antigen) producer cells obtained from a fully validated master cell bank (Chawla et al., 2019).

### 1.2 Studies on the mechanisms-of-action (MOA) of DeltaRex-G

i) Treatment of MG-63 and MNNGHOS cancer cells with DeltaRex-G *in vitro* induced an arrest of cancer cells in G1 phase, apoptosis of cancer cells and inhibition of cancer cell growth (Skotzko et al., 1995); ii) In a nude mouse model of osteosarcoma, intratumoral injections of DeltaRex-G induced blockade of the cell cycle in G1 phase, reduction in mitotic index, and shrinkage of tumors in DeltaRex-G-treated mice (Chen et al., 1997); iii) In a nude mouse model of pancreatic cancer, tail vein injections of DeltaRex-G induced down regulation of the CCNG1 protein, apoptosis of cancer cells and associated proliferative vasculature and shrinkage of tumors (Gordon et al., 2001); iv) Treatment of a patient with pancreatic adenocarcinoma metastatic to liver with DeltaRex-G intravenously and subsequent resection of residual liver nodule showed accumulation of immunoreactive vector particles (gp70 *env*) within the tumor microenvironment (TME), apoptosis of cancer cells, tumor associated microvasculature and tumor associated fibroblasts (Hall et al., 2010); v) Treatment of several pancreatic cancer patients with DeltaRex-G intravenously and subsequent resection of residual tumors showed immune cell trafficking in the TME consisting of CD35^+^ dendritic cells, CD4^+^ helper T cells, natural killer cells and CD8^+^ killer T cells. The preponderance of tumor-infiltrating lymphocytes in the TME supports the potential for utilizing DeltaRex-G in combination with immune-modulatory agents (Chawla et al., 2010; Dy et al., 2018).

### 1.3 Survival studies with DeltaRex-G therapy

Survival analysis of ninety-nine patients who received DeltaRex-G with or without DeltaVax, a tumor targeted retrovector encoding a granulocyte-macrophage colony stimulation factor (GM-CSF) gene showed 5% 10-year overall survival rate for patients who received DeltaRex-G alone, and 18.8% for DeltaRex-G + DeltaVax (Liu et al., 2021). These patients were refractory to chemotherapy and had metastatic disease: sarcoma (*n* = 4), pancreatic adenocarcinoma (*n* = 1), breast cancer (*n* = 2) and b-cell lymphoma (*n* = 1).

### Fast track designation, orphan drug designations and accelerated approval for DeltaRex-G

i) A US-based Phase 1/2 study using DeltaRex-G for advanced sarcoma showed a median overall survival (OS) of 11.5 months, a 1-year survival rate of 38.5%, and a 2-year survival rate of 31% (Chawla et al., 2016). These results gained orphan drug designation for soft tissue sarcoma and osteosarcoma; ii) A US-based Phase 1/2 study using DeltaRex-G for advanced pancreatic cancer showed a median survival of 9.2 months and a 1-year survival rate of 33% (Chawla et al., 2019). These results gained fast track designation for DeltaRex-G as second line therapy for advanced or metastatic pancreatic adenocarcinoma (Chawla et al., 2019); and iii) Based on the results of Phase 1/2 studies using DeltaRex-G for advanced solid tumors, the Philippine FDA granted accelerated approval for DeltaRex-G for patients with solid malignancies and no other treatment option (Gordon et al., 2006; Gordon et al., 2007).

In 2010, the clinical development of DeltaRex-G (Formerly Rexin-G) came to a standstill, and it was not until 9 years later that DeltaRex-G was revived when long term cancer survivors with DeltaRex-G therapy were reported (Liu et al., 2021) and expanded access for DeltaRex-G was granted by the USFDA. Here, we report on the 3-year results of the on-going Expanded Access Program for DeltaRex-G sponsored by the Aveni Foundation, an IRS approved 501c) (3) public charity.

## 2 Patients and methods

Study 1 is entitled “Blessed: Expanded Access for DeltaRex-G for Advanced Pancreatic Cancer and Sarcoma (NCT04091295)”. Study 2 is entitled “Individual Patient use INDs for solid malignancies” for compassionate reasons.

### 2.1 Endpoints

For Study 1 (Blessed), the primary endpoint is overall survival; the secondary endpoints are disease control rate (complete response, partial response, stable disease), best overall response rate, incidence and grade of treatment-related adverse events. For Study 2, overall survival and incidence and grade of treatment-related adverse events are the endpoints.

### 2.2 Eligibility criteria

For Study 1, Male or female patients ≥10 years of age and up to 40 patients with advanced sarcoma or pancreatic adenocarcinoma of any ethnicity will be treated. Patients with advanced metastatic pancreatic cancer who have received systemic therapies such as FOLFIRINOX and gemcitabine + albumin-bound paclitaxel; patients with metastatic sarcoma who have disease progression after two or more lines of systemic treatments and not amenable to surgical resection or radiotherapy; specifically for osteosarcoma: have disease progression after high dose methotrexate, cisplatinum, doxorubicin and ifosfamide; for soft tissue sarcoma: have disease progression after doxorubicin + ifosfamide/mesna, gemcitabine, docetaxel, dacarbazine, trabectedin, pazopanib, eribulin; patient who is intolerant to or declines available therapeutic options after documentation that patient has been informed of the available therapeutic options. For Study 2, individual patients of any age with solid malignancies will be treated using individual patient use Investigational New Drug (IND) applications.

### 2.3 Treatment schedule

For both studies, patients will receive DeltaRex-G at 1-3 x 10^11^ cfu i.v. over 30–45 min, three x a week for 3 weeks with a one-week rest period, until significant disease progression or unacceptable toxicity or death occurs. Complete blood counts, serum chemistry will be done every week; computerized tomography/magnetic resonance imaging will be done every 8–12 weeks. Of note, DeltaRex-G may evoke an inflammatory “flare” reaction in tumors causing an increase in the size of target lesions and/or non-target lesions or causing occult lesions to appear by radiologic imaging. In a previous study, a number of patients subsequently achieved either a complete response (CR), partial response (PR) or stable disease (SD) with continued DeltaRex-G treatment. Therefore, the following conditions for continuing therapy in the presence of radiologic progressive disease include: 1) There is clinical benefit, 2) There is no serious treatment related toxicity.

### 2.4 Statistical analysis

Patient characteristics, efficacy and safety analysis will use descriptive statistics and are hypothesis generating for planning Phase 2 studies. When possible, the STATA software will be used to generate Kaplan Meier plots, median progression freee survival (PFS) and overall survival (OS).

## 3 Results

### 3.1 Patient characteristics

Seventeen patients were enrolled, nine sarcoma, two pancreatic adenocarcinoma, one non-small cell lung cancer, two breast carcinoma, one prostate cancer, one cholangiocarcinoma and one basal cell carcinoma and actinic keratosis. Three patients were enrolled in Study 1 and 14 patients were enrolled in Study 2. Eleven of 17 enrolled patients were treated with DeltaRex-G monotherapy or in combination with FDA approved cancer therapies. Tables 1, 2 show patient characteristics.

**TABLE 1:** Patients enrolled in studies 1 and 2, according to race and gender.

Gender	White, not of hispanic origin	Black, not of hispanic origin	Hispanic	Asian, or pacific islander	Unknown	Total
Male	4	1	2	2	0	9
Female	6	0	0	2	0	8
Total	10	1	2	4	0	17

**TABLE 2:** Patients enrolled in studies 1 and 2, according to age group and gender.

Gender	11–28	29–39	40–50	51–61	62–72	73–90	Total
Male	1	2	1	3	-	2	9
Female	3	2	1	1	-	1	8
Total	4	4	2	4	-	3	17

### 3.2 Efficacy Analysis

Of the 12 treated patients, 5 (42%) are alive 15–36 months from DeltaRex-G treatment initiation. Two patients with early-stage triple receptor positive or triple receptor negative breast cancer who received DeltaRex-G as adjuvant/first line therapy are alive in complete remission 23 and 16 months after DeltaRex-G treatment initiation respectively; three patients with metastatic chordoma, chondrosarcoma and advanced basal cell carcinoma are alive 36, 31, and 15 months after DeltaRex-G treatment initiation respectively. Table 3 is a listing of patients surviving, their diagnosis, duration of current status and overall survival, and current therapy.

**TABLE 3:** Listing of surviving patients, clinical status, and current therapy.

Patient	Diagnosis	Previous therapy before DeltaRex-G	Clinical status and overall survival	Current therapy
1	Chordoma	Surgery, radiation, therapy, nab-sirolimus + nivolumab, trabectedin + nivolumab + talimogene laherparepvec	Progressive disease 36 months	Chemotherapy
2	Chondrosarcoma	Surgery, toripalimab, nab-sirolimus + nivolumab, gemcitabine + doxorubicin + docetaxel	Stable disease 31 months	Pazopanib
3	Early- stage ER + PR + HER2+ invasive breast carcinoma	Partial mastectomy	Complete remission 23 months	Letrozole
4	Early- stage triple receptor negative invasive breast carcinoma	Partial mastectomy	Complete Remission 16 months	None
5	Basal cell carcinoma and actinic keratosis	MOH surgery, diclofenac	Partial response 15 months	Diclofenac

### 3.3 Safety analysis

There were no treatment-related adverse events reported. Table 4 shows the adverse events unrelated to the study drug, and Table 5 is a listing of deaths, diagnosis, duration of survival and attribution to DeltaRex-G study drug.

**TABLE 4:** Adverse events unrelated to DeltaRex-G study drug by severity grade (*n* = 12).

Adverse event	Grade 1	Grade 2	Grade 3	Grade 4
Leukocytosis	2 (17%)			
Anemia	1 (8%)	1 (8%)	1 (8%)	
Nosebleed	1 (8%)			
Thrombocytopenia			1 (8%)	
Neutropenia			1 (8%)	
Irregular rate	1 (8%)			
Tachycardia		2 (17%)	2 (17%)	
Blurred vision		1 (8%)		
Abdominal distention			2 (17%)	
Ascites			3 (27%)	
Pain	1 (8%)	4 (33%)	3 (27%)	
Fatigue			4 (33%)	
Anxiety		1 (8%)		
Histoplasmosis, brain		1 (8%)		
Elevated Alk Phos		3 (25%)	3 (25%)	
Increased AST	2 (17%)	2 (17%)		
Increased ALT		2 (17%)		
Anorexia		1 (8%)	2 (17%)	
Weight loss			3 (25%)	
Hyponatremia		3 (25%)		
Hypocalcemia		1 (8%)		
Hypercalcemia		1 (8%)		
Hypoalbuminemia			1 (8%)	
Inc. LDH		1 (8%)	1 (8%)	
Left AKA	1 (8%)			
Cord compression				1 (8%)
Neck stiffness		1 (8%)		
Leg edema			1 (8%)	
Headache	1 (8%)			
Foot drop			1 (8%)	
Confusion			1 (8%)	
Wheezing		1 (8%)	1 (8%)	
Dyspnea			2 (17%)	
Decreased breath sounds			2 (17%)	
Nasal congestion		1 (8%)		
Nasal swelling/obstruction			1 (8%)	

**TABLE 5:** Listing of deaths, diagnosis, overall survival, cause of death and attribution.

Patient #	Diagnosis (Metastatic)	Previous therapy before DeltaRex-G	Overall survival, weeks	Cause of death	Attributable to DeltaRex-G?
1	Osteosarcoma	Cisplatin + Doxorubicin + MTX, surgery, Cisplatin + Doxorubicin + MTXifosfamide + etoposide, gemcitabine + docetaxel, sorafenib + everolimus, multiple resections, nab-sirolimus + nivolumab, ICE, radiation therapy, cabozantinib, trabectedin + nivolumab, aldoxorubicin	2	Disease progression	N
2	Pancreatic Adenocarcinoma	Folforinox, gemcitabine + nab-paclitaxel	13	Disease progression	N
3	Osteosarcoma	cisplatin + doxorubicin + methotrexate, surgery, cisplatin + doxorubicin + methotrexate, dinutuximab + GMCSF, nab-sirolimus + nivolumab, high dose ifosfamide, radical resection, aldoxorubicin	22	Disease progression	N
4	Desmoplastic Small Cell Tumor	VAC-IE, pazopanib, radiation therapy, trabectedin	8	Disease progression	N
5	Non-small Cell Lung Cancer	8 cycles carboplatin + pemetrexed + pembrolizumab	9	Disease progression	N
6	Osteosarcoma	methotrexate+ Li-Fraumeni Syndrome doxorubicin + cisplatin, ifosfamide, surgery, pazopanib, mifamurtide	27	Disease progression	N
7	Rhadomyosarcoma	vincristine + dactinomycin + cyclophosphamide, cincristine + irinotecan, temsirolimus, radiation therapy, oral cyclophosphade + vinorelbine, ifosfamide + etoposide, palbociclib + cyclophosphamide + topotecan, EGFR CAR-T, copanlisib	22	Disease progression	N

## 4 Discussion

More effective and less biologically toxic therapies are needed for patients with advanced cancers. Unfortunately, many therapies have failed during development, in part because they do not address the fundamental hallmarks of transformed cancer cells, nor the proximal oncogenic drivers of chemotherapy resistance, nor the progressive adaptations and oncogene addictions of malignancy. Our molecular genetic studies of the pivotal cyclin G1 (CCNG1) oncogene—conducted in the crucible of clinical oncology and analyzed in the context of intractable end-stage metastatic disease—provide new insights into the proximal and accessible Cyclin-G1/Cdk/Myc/Mdm2/p53 Axis of cell cycle activation and sustained cancer cell survival: revealing key “competence factors” and stem cell survival pathways, while identifying the cyclin G1 (CCNG1) oncogene as a strategic locus and clinical target for metastatic cancer control (Kim et al., 2017; Al-Shihabi et al., 2018; Gordon et al., 2018). Delivered intravenously as a tumor-targeted gene therapy expression vector, DeltaRex-G targets the collagenous extracellular matrices (anaplastic Signatures) of proliferative tumors, inducing cellular apoptosis in a broad spectrum of cancers. As an interventional gene targeted therapy for advanced metastatic cancers, repeated infusions of DeltaRex-G: i) may be as effective if not superior to currently used toxic chemotherapy drugs because it addresses a common defect and vulnerability in all cancers, i.e., uncontrolled cell cycling; and ii) is uniquely safe and maintains quality of life—which patients frequently suffer from during chemotherapy.

Previous data analysis indicated the feasibility, safety, and efficacy of tumor-targeted gene delivery *in vivo*, represented by the cytocidal gene vector DeltaRex-G—administered in clinical protocols, with or without an immuno-stimulatory tumor-targeted vaccine (DeltaVax: expressing GM-CSF), has induced prolonged (>10 years) sustained remissions in cancer patients presenting with advanced chemotherapy-resistant solid and hematologic malignancies—plausibly due to its combined properties of safety, efficacy, repeatability of infusions, proliferative cell selectivity, and tumor immune cell modulation. While the “curative potential” of precision targeted genetic medicine necessarily remains an academic question, it is clear that these initial long-term cancer (>10 years) survivors represent a major milestone in both cancer gene therapy and immunotherapy (Kim et al., 2017; Al-Shihabi et al., 2018; Dy et al., 2018; Liu et al., 2021).

DeltaRex-G is an off-the-shelf intravenous biologic and precision medicine for advanced cancers: an immunologically stealth retrovector displaying a tumor-Signature-seeking collagen-matrix-binding decapeptide on its gp70 Env protein, designed for binding to anaplastic collagenous proteins of the tumor microenvironment (TME). When injected i.v., the DeltaRex-G nanoparticles (∼100 nm in diameter) seek out and accumulate within the TME, in the vicinity of cancer cells, proliferating tumor-associated fibroblasts (TAFs) of tumor stroma, and endothelial cells of tumor-associated neovasculature. The vector then enters the target cell and delivers its cytocidal CCNG1 inhibitor gene (payload) into the nucleus of rapidly dividing cancer cells, TAFs and neoangiogenic cells, causing cell death *via* apoptosis-mediated pathways (Morse et al., 2021). Figure 1 shows an artist’s illustration of the DeltaRex-G retrovector, depicting its integral tumor-targeting function and the basic molecular mechanisms enforcing the expression of a cytocidal dominant-negative (dnG1) trans-gene, which blocks CCNG1/cyclin G1-dependent pathways of cell activation, proliferative competence, and survival functions.

**FIGURE 2:**
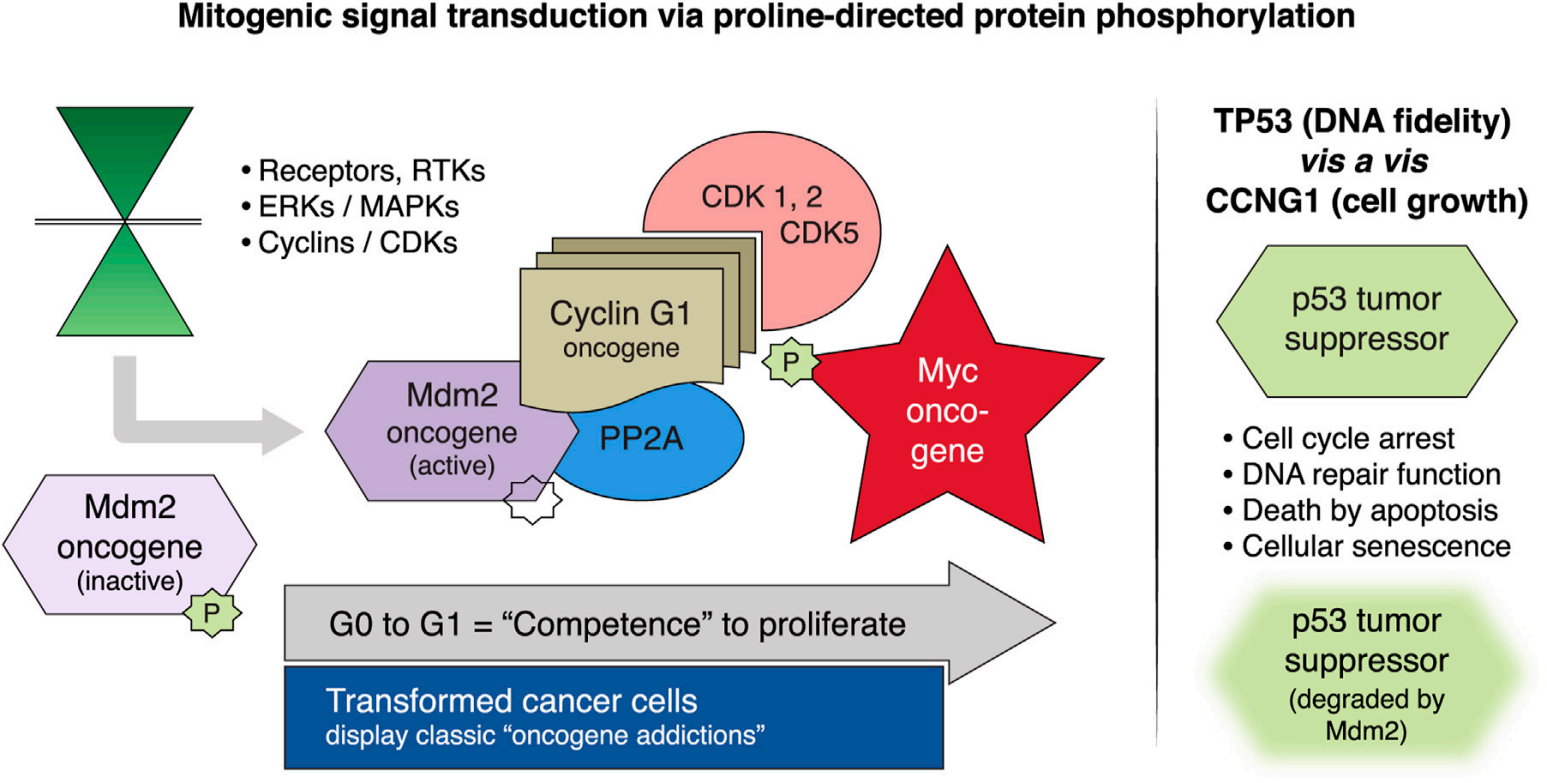
Mitogenic signaling pathways focused on the human cyclin G1 (*CCNG1* protooncogene). Left panel, MAPKs/ERKs and CDK complexes control the progressive phases of the cell division cycle. *CCNG1* physically binds to the PP2A to activate a key regulatory oncoprotein, Mdm2. The Mdm2 oncoprotein forms a physical complex with the p53 tumor suppressor, thus inactivating its tumor suppressor function, while also acting as a specific E3 ubiquitin ligase responsible for the ubiquitination and degradation of the p53 tumor suppressor protein. This dephosphorylation event is CCNG1-dependent. *CCNG1* also activates CDK5 and CDK1/2 to target/activate the c-Myc oncoprotein. Right panel, TP53 tumor suppressor functions are presented as opposite to the *CCNG1* growth-promoting function. *CCNG1*, cyclin G1; RTK, receptor tyrosine kinase; MAPKs, mitogen-activated protein kinases; ERKs, extracellular-signal-regulated kinases; CDK, cyclin-dependent kinase; PP2A, serine/threonine protein phosphatase subunit designated 2A; Mdm2, mouse double minute 2 homolog; TP53, tumor protein p53 (Morse et al., 2021).

Mechanistically, the cybernetic/regulatory theme of proline-directed protein phosphorylation links the intricate protein kinase cascades of mitogenic signal transduction to the fundamental biochemistries of cellular activation, transcriptional competence, cell survival, chemotherapy resistance, and cell fate. Proline-directed (site-specific ser and thr) phosphorylation proceeds from the activation of various extracellular receptor tyrosine kinases (RTKs) through an array of intermediate ERKs/MAPKs to the executive cyclins and cyclin-dependent kinases (CDKs), which essentially govern cellular competence, survival, and genome stability during the cell cycle. Foremost in terms of proximal effectors is the cyclin G1 protein encoded by the CCNG1 proto-oncogene; as shown in Figure 2, along with the main constellation of executive interacting oncoproteins and tumor suppressor proteins identified as the cyclin G1/Cdk/Myc/Mdm2/p53 Axis of cell cycle activation, cell competence, and survival (Kim et al., 2017; Al-Shihabi et al., 2018; Gordon et al., 2018). Biochemically, cyclin G1, serves as an activating and targeting subunit of its cognate cyclin-dependent kinase(s) (Cdk1, 2, or 5), which activates and stabilizes the c-Myc oncoprotein on the one hand; while cyclin G1 additionally recruits the phosphatase PP2A to selectively de-phosphorylate and activate Mdm2 (a p53 ubiquitin ligase), which thereby degrades p53 and its tumor suppressor function and leads to error-prone DNA replication and repair that is so often associated with progressive metastatic disease. Following c-Myc activation by site-specific phosphorylation on serine-62, the c-Myc competence factor is further stabilized by physical association with PIN1, a proline-dependent phosphorylation-directed cis/trans isomerase, which further enhances and directs the transcriptional and oncogenic activities c-Myc (Cohn et al., 2020).

The proximal oncogenic role of cyclin G1 (CCNG1) is supported by viral subversion of the locus—by the hepatitis B virus (Hbx protein)—and by molecular screening studies of hepatocarcinogenesis, where miR-122 was identified as a pivotal regulatory microRNA that directly targets and suppresses CCNG1 expression and is commonly lost with disease progression. Moreover, a number of newly discovered oncogenic long non-coding RNAs, including lncRNA HOTAIR, act by molecular and epigenetic means to suppress miR-122 expression, thus unleashing cyclin G1 expression from stringent inhibitory control (Cheng et al., 2018). Importantly, a case report of mutational activation involving the CCNG1 promoter was recently identified in a breast cancer patient suffering (subsequently) from chemotherapy-related acute myeloid leukemia—which suggested overexpression of the cyclin G1/Cdk2/c-Myc as drivers, while recognizing DeltaRex-G as a prospective and rational therapy in myeloid tumors with typically pore prognosis (Xiao et al., 2020). In terms of clinical oncology, the demonstrated utility of tumor-targeted DeltaRex-G as monotherapy are two-fold: i) providing a singular and decisive (i.e, proximal and effective) point of cell cycle control for therapeutic intervention; and ii) providing a rational broad-spectrum therapeutic option for otherwise intractable cancers.

## 5 Conclusion

Taken together, the clinical data from Expanded Access for DeltaRex-G suggest that i) DeltaRex-G may evoke tumor growth stabilization after failing standard chemotherapy, ii) DeltaRex-G may act additively or synergistically with standard chemotherapy and/or tumor-targeted immunological therapies, and iii) Adjuvant/first line therapy using DeltaRex-G may be authorized by the USFDA in patients with early-stage invasive carcinoma of the breast or patients with other solid malignancies who refuse toxic chemotherapy.

## Data Availability

The raw data supporting the conclusions of this article will be made available by the authors, without undue reservation.
